# Post-activation performance enhancement does not occur following a large hand-paddles and parachute-resisted warm-up routine in collegiate swimmers

**DOI:** 10.3389/fspor.2023.1244168

**Published:** 2023-11-24

**Authors:** Santiago Soares Bufalo, Gabriel Fontanetti, Renan Vieira Barreto, Gabriel Rocha Benazzi, Rubens Correa Junior, Victor Marangoni, Natália de Menezes Bassan, Benedito Sérgio Denadai, Camila Coelho Greco, João Paulo Vilas-Boas, Leonardo Coelho Rabello de Lima

**Affiliations:** ^1^ Human Performance Laboratory, Department of Physical Education, São Paulo State University, Rio Claro, Brazil; ^2^ Center of Research, Education, Innovation, and Intervention in Sport and LABIOMEP-UP, Faculty of Sport, University of Porto, Porto, Portugal; ^3^ School of Physical Education and Sport of Ribeirão Preto, University of São Paulo, Ribeirão Preto, Brazil

**Keywords:** swimming, post-activation performance enhancement, warm-up, priming, freestyle, kinematics

## Abstract

Our aim was to investigate if using a warm-up routine that included parachute-resisted sprints with large hand-paddles improves 50 m freestyle performance in trained collegiate swimmers. Twelve swimmers (23.9 ± 2.2 years, 179 ± 7 cm, 77.1 ± 10.6 kg) participated in the study and completed two 50-m freestyle races, each preceded by a different warm-up routine, either control (CON) or experimental (EXP). The warm-up routines consisted of 500 m of swimming at self-selected speed, followed by four 10 s sprints with 1 min rest intervals. During EXP, sprints were performed using large hand-paddles and a swimming parachute, while during CON, sprints were performed freely. Performance and technique were assessed during the 50 m freestyle races. We found no significant differences in 25- and 50 m performance times (CON: 12.6 ± 0.8 vs. EXP: 12.5 ± 0.8 s, ES = 0.125; and CON: 26.8 ± 1.6 vs. EXP: 26.7 ± 1.7 s, ES = 0.06, respectively) between the two conditions. Mean stroke length (CON: 2.04 ± 0.21 vs. EXP: 2.02 ± 0.22 m·cycle^−1^, ES = 0.09), stroke frequency (CON: 55.4 ± 5.3 vs. EXP: 56.3 ± 5.2 cycles s^−1^, ES = 0.17), and propulsive time (CON: 0.62 ± 0.07 vs. EXP: 0.61 ± 0.06 s, ES = 0.15) were also not different between conditions. It is possible that the CON warm-up routine induced the priming effects that lead to PAPE, or that the EXP warm-up routine primed the athletes further but also induced greater fatigue, resulting in no significant effects on swimming performance. Our findings suggest that parachute-resisted sprints with hand-paddles during warm-up do not enhance 50 m freestyle swimming performance in trained collegiate swimmers. Coaches and practitioners should consider exploring different warm-up protocols to identify what works best for their athletes.

## 1. Introduction

The 50 m freestyle swimming race is one of the fastest events in competitive swimming, relying heavily on an athlete’s explosive force and swimming technique (1, 38). In fact, a classical study by Hawley et al. (2) demonstrated that upper- and lower-limb muscle power, as assessed during Wingate Anaerobic Tests, were significantly correlated with 50 m swimming speed (*r* = 0.63 and 0.76, respectively). More recent data from Loturco et al. (3) support this idea, as they found strong correlations (*r* = −0.72 and −0.76, respectively) between the rate of force development and impulse during tethered swimming and 50 m freestyle swimming time. These findings highlight the importance of both muscle power and swimming technique in achieving optimal performance in the 50 m freestyle swimming race.

Explosive force is a key component of human performance that can be developed through strength training (4). However, there are also strategies to acutely enhance muscle power that can be used in conjunction with strength training. One such strategy is post-activation performance enhancement (PAPE) (5–7). PAPE has been extensively studied in the past two decades, particularly in relation to land-based activities like jumping (8, 9) and sprinting (10, 11). For these types of activities, it is well-established that performing pre-activation activities involving heavy weightlifting a few minutes prior to explosive muscle actions leads to improved performance (10–13). The magnitude of this effect can be influenced by several factors, including strength, sex, level of training, and the protocol of the pre-activation activity (including the amount of resistance, number of sets, and time between the pre-activation and the potentiated action) (7, 14). Despite being consistently investigated in land-based activities, the occurrence of PAPE in swimming is still being studied and has produced conflicting results (15, 16).

To the best of our knowledge, the study by Kilduff and colleagues (17) was the first to investigate the effects of PAPE in swimmers. They reported that performing one set of heavy-weight squats approximately 8 min prior to a 15 m swim start led to improvements in peak vertical and horizontal forces applied to the block, but no improvements in 15 m time compared to traditional warm-up. Other studies have also investigated the effectiveness of different pre-activation protocols in enhancing start and swimming performance. These protocols have involved dryland traditional resistance exercise (for either upper and lower limbs or a combination of both) (18–25), dryland lower-limb flywheel exercise (19), combined lower- and upper-limb flywheel exercise (23), tethered swimming (26, 27), and parachute-resisted swimming (28).

While the studies mentioned have contributed to understanding swimming PAPE, most of them utilized methods that are not practical for everyday training or competitions due the sizes, weights, and ease of transportation of the equipment involved or the complexity of the logistics of the pre-activation protocols. To our knowledge, only Barbosa et al. (28) investigated the effects of a parachute-resisted pre-activation protocol on swimming propulsive force but, although they did assess swimming performance, time to completion of simulated races was not investigated. Thus, there is a gap in the literature regarding ecologically valid in-water pre-activation methods that can enhance swimming performance using accessible equipment and infrastructure.

Schnitzler et al. (29) showed that parachute-resisted swimming results in greater drag force and, consequently, greater propulsive force required to sustain the same swimming velocity. Additionally, swimming with hand paddles was shown to increase average stroke force and impulse during tethered swimming (39). Considering that responsiveness to PAPE depends on the amount of resistance applied during the pre-activation exercise (7), parachute-resisted swimming using large hand-paddles appears to be a good, ecologically valid, and practical strategy to induce such phenomenon in sprint swimmers. Therefore, we aimed to determine warming up with parachute-resisted sprints with large hand-paddles improves 50 m freestyle swimming performance. We hypothesized that such warm-up strategy would improve performance in a 50 m swim by increasing stroke frequency.

## 2. Material and methods

### 2.1. Participants

Thirteen male collegiate swimmers participated in the study, all of whom competed at college and regional levels and were in the post-competitive phase of the season. Participants in the present study meet the criteria for inclusion in tier 2 of the participant classification framework proposed by McKay et al. (39) (i.e., local-level representation, regularly training ∼3 time per week, and training with a purpose to compete). In a preliminary qualifying session, all athletes were required to perform a 50 m freestyle race starting from the block in less than 28 s to be included in the study. One subject failed to meet this requirement and was excluded from the sample, leaving a final sample size of 12 swimmers. The mean age, height, body mass, and body mass index of the participants were 23.9 ± 2.2 years, 179.1 ± 7.1 cm, 77.1 ± 10.6 kg, and 23.9 ± 2.2 kg.cm^−2^, respectively. The subjects were instructed to maintain their usual dietary habits and to arrive at the experimental sessions at least two hours after a full meal. The study protocol was approved by the institutional ethics review board and conducted in accordance with the Helsinki declaration for the use of humans as research subjects. All participants signed an informed consent form before undergoing any experimental procedures.

### 2.2. Experimental design

The experiment was conducted over two sessions, which took place at the same time of day and were separated by at least two and at most seven days. During the first session, the subjects provided written consent and had their anthropometric parameters assessed before performing two 50 m freestyle race preceded by self-selected warm-up routine. The races were separated by 40 min. These races were used as an exclusion criterion, as the finishing times had to be less than 28 s for inclusion. At this point, all dependent variables of the study, including 25- and 50 m swimming times, stroke frequency, stroke length, and propulsive time, were assessed to determine the reliability of the measurements.

After the 50 m freestyle race, the subjects had a 10 min recovery period and were then familiarized with parachute-resisted swimming with large hand-paddles. They performed submaximal 15 m sprints while wearing large hand-paddles and a belt attached to a commercially available swimming parachute (Swimming Parachute, Kytec, USA).

During the second session, the subjects performed two 50 m freestyle races preceded by either a control (CON) or an experimental (EXP) warm-up in a cross-over design. The order of the warm-ups was randomized electronically (Excel, Microsoft, USA), and the races were separated by a 40 min rest interval. The warm-up routines consisted of 500 m of swimming at a self-selected velocity, followed by four maximal 10 s sprints with 1 min rest intervals. During the EXP warm-up, sprints were performed using large hand-paddles and a swimming parachute, while during the CON warm-up, sprints were performed freely. All procedures were conducted in a 25 m swimming pool with clear water.

### 2.3. Procedures

The CON warm-up in this study involved the swimmers completing 500 m of self-selected speed swimming, followed by a two-minute rest period and four 10 s maximal sprints with 1 min rest intervals. During the sprints, the swimmers were not allowed to use the wall of the pool to push off and had to rely solely on their stroke and kicking movements for propulsion. The EXP warm-up was similar to CON, but the swimmers wore the same large hand-paddles and swimming parachute that were used in the familiarization session. A previous study by Schnitzler et al. (29) demonstrated that using the same parachute with the same configuration increased drag force significantly, which led to increased maximal stroke propulsive force and stroke propulsive impulse. Prior to each sprint, one of the examiners held the swimming parachute in place to ensure that there was a constant and uniform provision of extra drag from the beginning to the end of each effort.

Six minutes after completing both warm-ups, the subjects performed a 50 m freestyle race starting from the blocks. The start was self-determined to eliminate the influence of reaction time. The races were recorded from both under- and overwater perspectives in the sagittal plane using a custom-made trackless cart. The cart was designed to run alongside the pool with high-definition (720p) and high-frequency (120 Hz) cameras (Hero 4, GoPro, USA) firmly attached to underwater and overwater arms. Both cameras were synchronized using a flashing light before each experimental procedure.

Swimming performance was measured by the time it took each swimmer to complete the first 25 m lap and the full 50 m race. Because the race start was self-determined, the chronometer was activated when both feet of the swimmers left the block. The time to complete the first 25 m was recorded as the moment the first foot of the swimmer touched the wall during the turn. The time to complete the full 50 m race was recorded as the moment the first hand of the swimmer touched the wall at the end of the second lap.

The swimming technique of the participants was evaluated by analyzing their kinematics during the 50 m freestyle race. The analysis included stroke frequency, stroke length, and stroke propulsive time. To ensure accurate results, the first 10 meters and last 2.5 m of each lap were excluded from the analysis, as these sections may have been affected by starting off the blocks or adaptive swimming before the turn or finish. Markings on the pool floor and floating lanes were used to identify the appropriate range for analysis. Kinematic analysis was performed by an experienced analyzer who was blinded to the experimental conditions, using a freely available software (Kinovea, Open Source). Stroke frequency (SF) was calculated based on the time taken to perform five strokes and expressed as cycles per minute (cycles.min^−1^). Stroke length (SL) was calculated by dividing swimming speed by SF, and expressed as meters per cycle (m.cycle^−1^). Stroke propulsive time was indirectly estimated for each stroke by measuring the time taken during phases B (pull) and C (push) of swimming. Phase B was considered to start after phase A (entrance and slide phase) ended and finished when the shoulder was flexed at 90°. Phase C was considered to start at the end of phase B (i.e., when the shoulder was flexed at 90°) and finished as soon as the hand left the water for the recovery phase (i.e., the start of phase D). The mean propulsive time for five consecutive strokes was used for analysis. For a more detailed description of the stroke phases, please refer to Bassan et al. (30).

### 2.4. Statistical analyses

The data collected was entered into an Excel spreadsheet and stored for further analysis. Statistical analysis was conducted using the Statistical Package for the Social Sciences (SPSS 18.0, IBM, USA). To ensure that the data was normally distributed, the Shapiro–Wilk’s test was performed. Differences in the dependent variables between the CON and EXP conditions were analyzed using two-tailed Student’s *t*-tests for paired samples. A *p*-value less than 0.05 was considered statistically significant. Due to the highly individualized nature of PAPE, the smallest worthwhile change (SWC) method was used to track meaningful changes. This method determines individual thresholds for changes by considering an effect size and the standard deviation for the variable in a given population (31). An effect size of 0.2 was used for analysis, and this value was multiplied by the standard deviations of the 25 m and 50 m times during the CON conditions to obtain SWC values of 0.16 and 0.33 s, respectively. Therefore, any differences (positive or negative) in the 25 m and 50 m times between the EXP and CON conditions that were greater than 0.16 and 0.33 s, respectively, were considered significant for individual analysis.

## 3. Results

**Figure 1:**
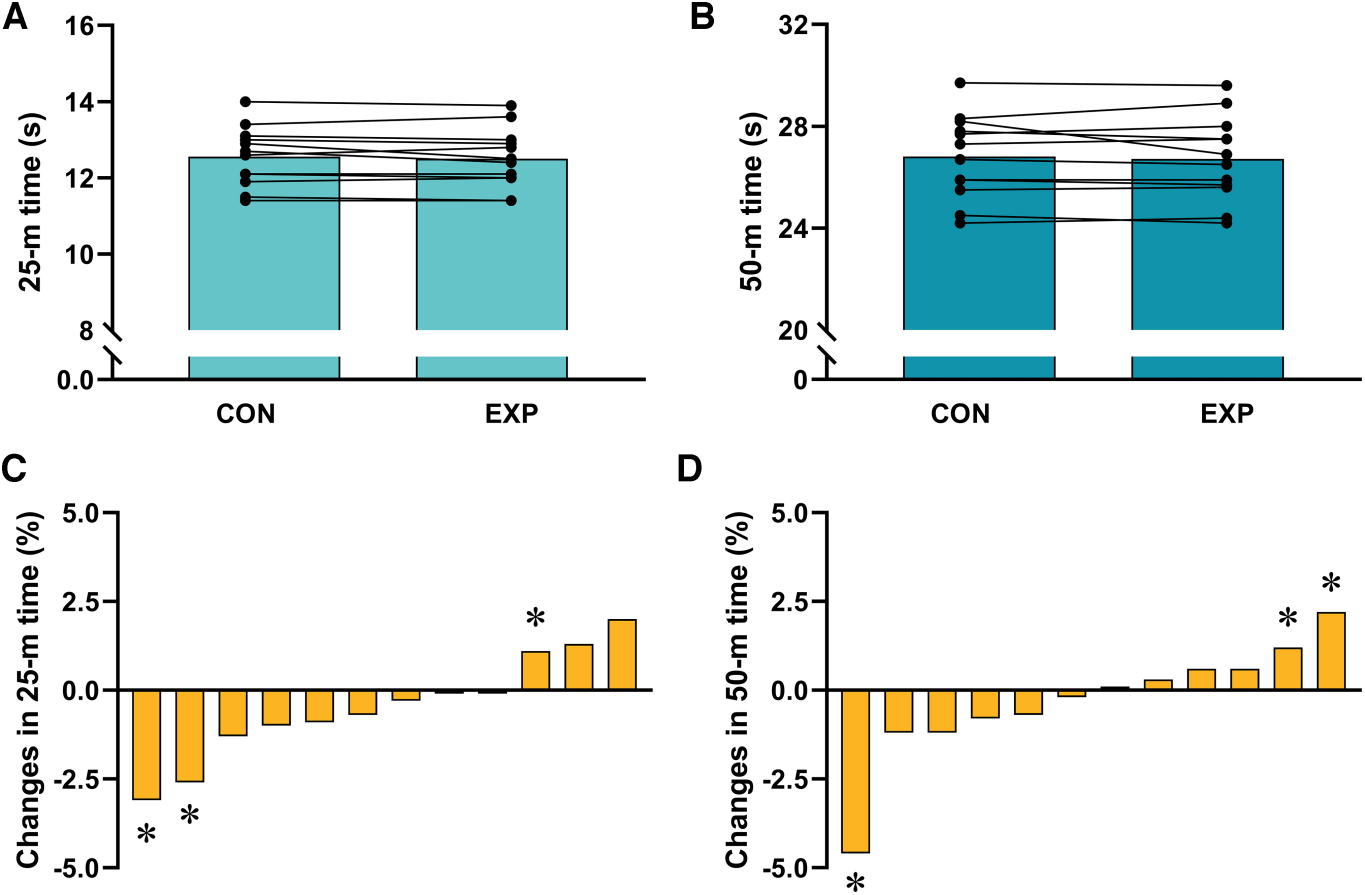
Swim times for 25- (**A**) and 50-m (**B**) in the control (CON) and experimental (EXP) conditions. Panels **C** and **D** represent individual changes in 25- (**C**) and 50-m (**D**) swimming times comparing CON and EXP warm-ups. *Changes from CON greater than the calculated smallest worthwhile change.

The reliability of the measurements was high, with intraclass correlation coefficients and confidence intervals of 0.93 (0.77–0.98), 0.91 (0.70–0.98), 0.94 (0.80–0.98), 0.91 (0.71–0.98), and 0.94 (0.78–0.98) for 25 m and 50 m times, stroke frequency, stroke length, and stroke propulsive time, respectively. Figure 1 displays the results for swimming performance. The 25 m swimming time did not differ significantly (*p* = 0.28) between the CON (12.6 ± 0.8 s) and EXP (12.5 ± 0.8 s) conditions (Figure 1A), and the same was true for the 50 m swimming time (CON: 26.8 ± 1.6 s; EXP: 26.7 ± 1.7 s; *p* = 0.54) (Figure 1B). Individual analyses using the SWC method showed that two subjects had decreased and one had increased their 25 m swimming times (Figure 1C), whereas one subject decreased and two increased their 50 m swimming times (Figure 1D).

**Figure 2:**
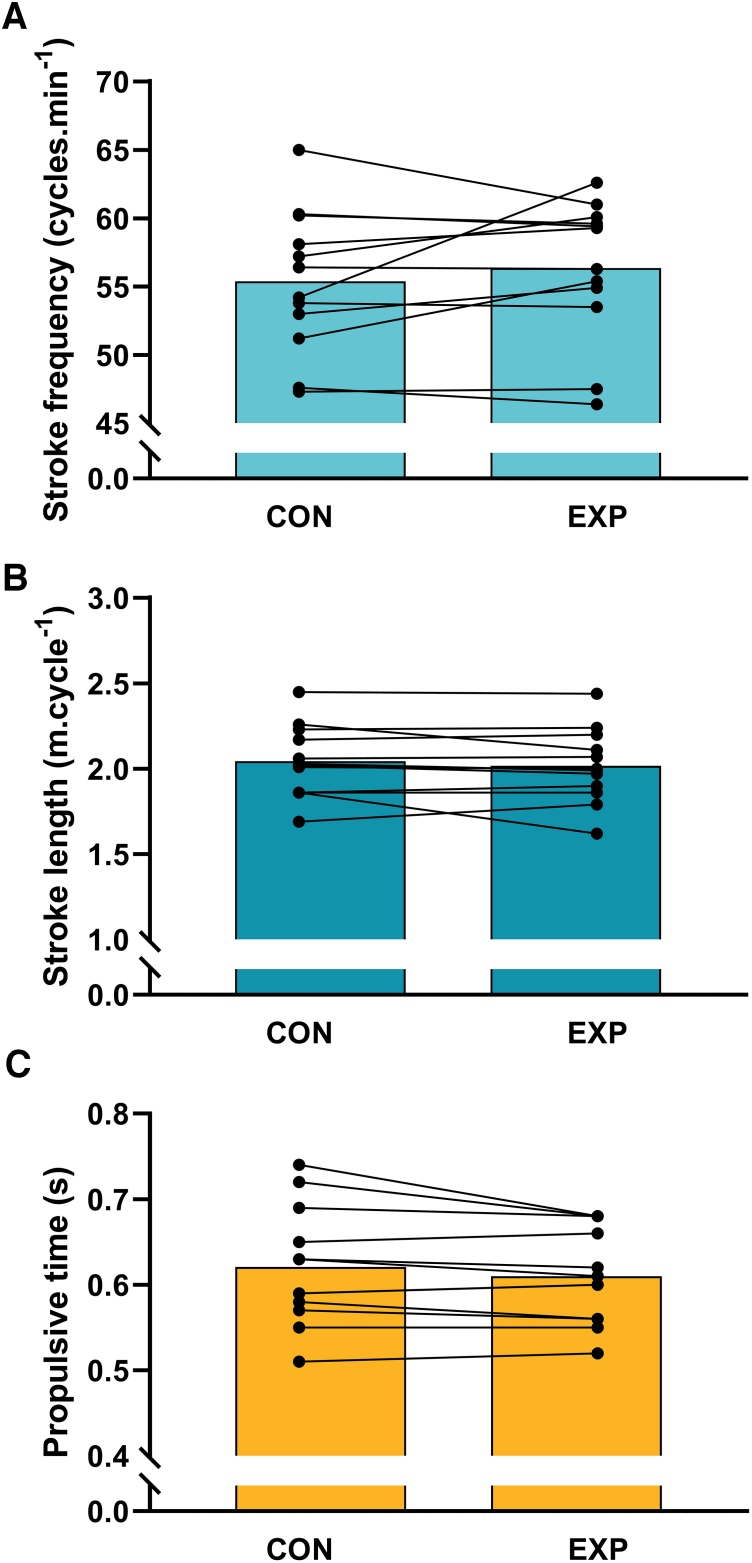
Swimming technique parameters (**A**: stroke frequency; **B**: stroke length; **C**: stroke propulsive time) in the control (CON) and experimental (EXP) conditions.

Figure 2 presents the swimming technique data, which showed that none of the assessed kinematic parameters were significantly affected by the different warm-up conditions. There were no significant differences in stroke frequency between the CON (55.4 ± 5.3 cycles.min^−1^) and EXP (56.3 ± 5.2 cycles.min^−1^) conditions (*p* = 0.32). Similarly, stroke length was not significantly different between the CON (2.04 ± 0.21 m.cycle^−1^) and EXP (2.02 ± 0.22 m.cycle^−1^) conditions (*p* = 0.33). Additionally, stroke propulsive time was not significantly different between the CON (0.62 ± 0.07 s) and EXP (0.61 ± 0.06 s) conditions (*p* = 0.06).

## 4. Discussion

The main goal of this study was to examine whether using large hand-paddles and performing parachute-resisted sprints as a warm-up could lead to improvements in swimming performance during a subsequent 50 m freestyle race, as well as changes in swimming technique. The hypothesis was that the additional drag and propulsion from these warm-up techniques would result in a performance enhancement. However, the results showed that there were no significant differences in performance between the experimental (EXP) warm-up and the traditional (CON) warm-up. Additionally, there were no observable differences in swimming technique between the two warm-up routines.

PAPE is most relevant for powerful actions, such as dryland sprints, jumps and, in the case of the present study, 50 m freestyle swim races, which are the most powerful swimming events in competitive swimming (1, 2). It can be used as a pre-competitive strategy to enhance performance during races, as well as during training sessions to improve their quality, which is commonly referred to as contrast training (32, 33).

Several factors have been suggested to influence responsiveness to PAPE, and it seems that training status plays a role in modulating the response to pre-activation activities. Studies indicate that trained and/or stronger individuals tend to respond better to dryland PAPE compared to their untrained or weaker counterparts (7, 14). For the present study, collegiate swimmers who could complete 50-m freestyle races in under 28 s were recruited. Although these times are not at the elite level, this exclusion criterion was implemented to ensure that all participants were experienced and capable of performing above the amateur level. As a result, the sample was relatively homogeneous, and more importantly, familiar with high-speed swimming training. This may have reduced the responsiveness bias associated with training status that is commonly observed in PAPE studies (7, 8).

The present study did not observe any PAPE response for the dependent variables investigated following the EXP warm-up. As far as we know, this is only the second study to investigate responsiveness to PAPE following parachute-resisted sprint swimming. Barbosa et al. (28) reported that a different configuration of parachute-resisted swimming (8 × 12.5 m sprints) resulted in compromised peak force and impulse but not the rate of force development during maximal tethered swimming. The authors discussed that the pre-activation protocol used in their study might have imposed excessive stress on the swimmers, resulting in a fatigued state during performance assessments. With this in mind, we have adjusted the EXP protocol of our study by reducing the number of sprints to modulate the stress imposed and allow for optimal recovery. Our findings suggest that the investigated warm-up protocol was not stressful enough to compromise performance, but it also did not appear to have been stressful enough to elicit the physiological mechanisms underpinning PAPE.

Several other studies investigating PAPE for swimmers have suggested using dryland exercises such as repeated jumps, squats, and pull-ups in the warm-up protocol (18–25). However, a frequent observation of these studies is that, although successful in improving swimming start performance (15, 23–25), the potentiating effect induced by dryland exercise does not appear to be transferred to swimming (20, 21, 23, 24). This often leads to improved start performance but no impact on overall race performance, which is mainly determined by swimming, as it accounts for the majority of the race time.

Our study’s results are similar to those of Abbes et al. (20), who found no changes in 50 m swimming performance for regional-level swimmers after a warm-up consisting of three 10 s maximal tethered swimming sprints. However, Hancock et al. (26) observed improvements in 100 m freestyle swimming performance in collegiate swimmers following four 10 m maximal tethered swimming sprints. Nonetheless, it is important to note that comparisons between studies should be made carefully, as tethered swimming differs from parachute-resisted swimming in terms of load control during sprints. Given our objective of proposing an ecologically valid warm-up strategy that could be used in everyday training sessions and competitive settings, tethered swimming was not a practical option.

The lack of difference in swimming performance and technique following the EXP warm-up in our study may be attributed to its similarity with the CON warm-up. The only variation between the two warm-ups was the intensity of the four 10 s sprints at the end of the routine. Since high-intensity efforts are usually included in sprinters’ warm-up routines, we chose to increase the stress imposed to muscles by adding a parachute and large hand-paddles to further increase drag force. Schniztler et al. (29) showed that using the same parachute led to a 12.6% increase in peak propulsive force and a 9.8% increase in propulsive impulse during constant-speed tethered swimming. In our study, we did not quantify the additional resistance imposed by the parachute, which can be considered a limitation. This quantification could have been used to compare the amount of imposed stress between the two warm-up routines. However, we speculate that a significant amount of stress was already provided during the sprint phase of the CON warm-up routine and that the additional drag imposed by parachute-resisted sprinting during the EXP warm-up may have been insufficient to enhance swimming technique and performance further.

It is generally agreed that using heavy loads, around 80%–90% of the one-repetition maximum (1RM), is the most effective way to induce PAPE during dryland exercises (7), with studies comparing light and heavy pre-activation protocols having shown that the former can produce greater improvements in jump height and repeated sprint ability (34, 35). While we acknowledge these findings, we do not think it would be appropriate to compare a heavy-load warm-up protocol, such as parachute swimming, with a warm-up routine that does not include high-intensity efforts like short-distance sprints, as these sprints are a common element of traditional warm-up protocols for sprint swimmers. Thus, we suggest that future studies should explore different experimental warm-ups to find the optimal pre-activation load that can trigger PAPE without causing performance-reducing fatigue.

The recovery interval between the pre-activation warm-up routine and performance assessment can have a significant impact on responsiveness to PAPE. In our study, we used a 6 min recovery interval, which has been shown to allow for fatigue recovery while also benefiting from the relatively short-lasting mechanisms underpinning PAPE (5, 7, 36). However, the ideal recovery interval for PAPE is highly variable and controversial, even among individuals with the same training background (37). It is possible that some subjects in our study may have responded to the EXP warm-up protocol, but the 6-minute recovery window was not optimal for them. One possible solution is to use a self-selected recovery interval, where individuals are allowed to choose the interval between the pre-activation and performance assessment by filling out a readiness scale. A recent study by Carmo et al. (36) demonstrated that this approach resulted in greater responsiveness to PAPE in trained individuals. This should be considered when designing future studies investigating PAPE.

## 5. Conclusions

The results of our study suggest that adding a swimming parachute and large hand-paddles to warm-up routines may not enhance 50 m freestyle swimming performance compared to traditional warm-ups. Coaches and practitioners are encouraged to experiment with different loads during warm-up routines to determine whether parachute-resisted sprints can improve their athletes’ swimming performance. It is also important to investigate the optimal recovery time between warm-up routines and swimming efforts. By doing so, coaches and practitioners can determine whether traditional warm-ups are already optimal for their athletes or if there are better warm-up routines to improve swimming performance.

## Data Availability

The original contributions presented in the study are included in the article/Supplementary Material, further inquiries can be directed to the corresponding author.
